# ContactJ: Lipid droplets-mitochondria contacts characterization through fluorescence microscopy and image analysis

**DOI:** 10.12688/f1000research.51900.1

**Published:** 2021-04-01

**Authors:** Gemma Martín, Marta Bosch, Elisenda Coll, Robert G. Parton, Albert Pol, Maria Calvo

**Affiliations:** 1 Advanced Optical Microscopy Facility. Scientific and Technological Centers, University of Barcelona, Barcelona, 08036, Spain; 2 Biomedicine Department. School of Medicine, University of Barcelona, Barcelona, 08036, Spain; 3 Cell Compartments and Signaling Group, Institut d'Investigacions Biomèdiques August Pi i Sunyer (IDIBAPS), Barcelona, 08036, Spain; 4 Institute for Molecular Bioscience (IMB), University of Queensland, Brisbane, Queensland, 4072, Australia; 5 Centre for Microscopy and Microanalysis, University of Queensland, Brisbane, Queensland, 4072, Australia; 6 Institució Catalana de Recerca i Estudis Avançats (ICREA), Barcelona, 08010, Spain

**Keywords:** Contact sites, Lipid Droplets, Mitochondria, Image Processing and Analysis, ImageJ, Fluorescence Microscopy, Bioimaging, Interactome

## Abstract

Lipid droplets (LDs) are the major lipid storage organelles of eukaryotic cells and together with mitochondria key regulators of cell bioenergetics. LDs communicate with mitochondria and other organelles forming “metabolic synapse” contacts to ensure that lipid supply occurs where and when necessary. Although transmission electron microscopy analysis allows an accurate and precise analysis of contacts, the characterization of a large number of cells and conditions can become a long-term process. In order to extend contact analysis to hundreds of cells and multiple conditions, we have combined confocal fluorescence microscopy with advanced image analysis methods. In this work, we have developed the ImageJ macro script ContactJ, a novel and straight image analysis method to identify and quantify contacts between LD and mitochondria in fluorescence microscopy images allowing the automatic analysis. This image analysis workflow combines colocalization and skeletonization methods, enabling the quantification of LD-mitochondria contacts together with a complete characterization of organelles and cellular parameters. The correlation and normalization of these parameters contribute to the complex description of cell behavior under different experimental energetic states. ContactJ is available here: https://github.com/UB-BioMedMicroscopy/ContactJ/tree/1.0

## Introduction

Lipid droplets (LDs) are the major lipid storage organelles of eukaryotic cells and together with mitochondria key regulators of cell’s bioenergetics. They supply essential lipids to produce signalling molecules, membrane building blocks, and the metabolic energy needed to survive during nutrient poor periods. ^1^

In order to achieve their functions, LDs communicate with mitochondria and other organelles (endoplasmic reticulum, endosomes, peroxisomes and vacuoles) forming membrane contact sites, ^2^ “metabolic synapses”, to ensure that lipid provision occurs where and when necessary. ^1^^,^^3^^,^^4^ Contact sites between these organelles have been described and characterized by transmission electron microscopy (TEM) as it resolves at the membrane scale where these contacts take place. ^2^^,^^5^ Whereas TEM allows accurate and precise characterization of contacts, their analysis on a large number of cells and conditions can become a long-term process. On the other hand, confocal fluorescence microscopy combined with advanced image analysis methods enable to extend contact analysis to hundreds of cells and multiple conditions.

Typically, in fluorescence microscopy, the contacts between cellular organelles, the organelle interactome, have been studied by colocalization or overlapping regions of the organelles masks ^6^ or measuring the fraction of intensity of other organelles near to LD. ^7^

In the present work, we describe a novel and straight image analysis method (ContactJ) to identify and quantify contact regions between LD and mitochondria in fluorescence microscopy images allowing the automatic analysis of hundreds of cells and multiple conditions. This image analysis workflow combines colocalization and skeletonization methods, enabling the detection of LD-mitochondria contacts together with a complete characterization of organelles and cellular parameters (morphometry and distribution). The correlation and normalization of these parameters contribute to the complex description of cells response under different experimental conditions such as metabolic or pathogenic states.

## Methods

### Sample preparation and imaging

Sample preparation and imaging have been previously described in detail. ^4^ Briefly, HEK293 cells were grown in fibronectin coated glass coverslips. Cells were fixed for 60 min in 4% paraformaldehyde, permeabilized in 0.15% Triton X-100 for 10 min, followed by blocking with 1% BSA (A7906, Sigma-Aldrich), 0.1% Tween in PBS for 15 min. Labeling was achieved by incubating cells for 1 hour at room temperature with rabbit polyclonal anti-TOM20 (1:500; ab186734, Abcam) diluted in blocking solution. Primary antibody was detected with donkey anti-rabbit IgG AlexaFluor 555 (A321094) from ThermoFisher Scientific, diluted 1:250 in blocking solution. Finally, cells were labeled with DAPI (1: 4000; ThermoFisher) and LDs were stained with BODIPY 493/503 (1:1000; Molecular Probes) for 10min at room temperature, washed twice with PBS and coverslips were mounted with Mowiol (475904; Calbiochem, Merck).

Imaging of LDs, mitochondria and nuclei was performed using a LSM880 laser scanning spectral confocal microscope equipped with an AxioObserver Z1 inverted microscope. DAPI, BODIPY 493/503, and Alexa Fluor 555 images were acquired sequentially using 405, 488 and 561 nm lasers, dichroic beam splitters, emission detection ranges of 415-480 nm, 500-550 nm and 571-625 nm, respectively, and the confocal pinhole was set at 1 Airy Unit (AU). Spectral detection was performed using 2 photomultipliers and 1 central GaAsP. Images were acquired in a 1024 × 1024 format, pixel size at 93 × 93 nm, and integration time of 0.51 microseconds. Sample preparation and image acquisition of TEM image from Figure 3a has been previously described in detail. ^5^

### Implementation

**Figure 1.**
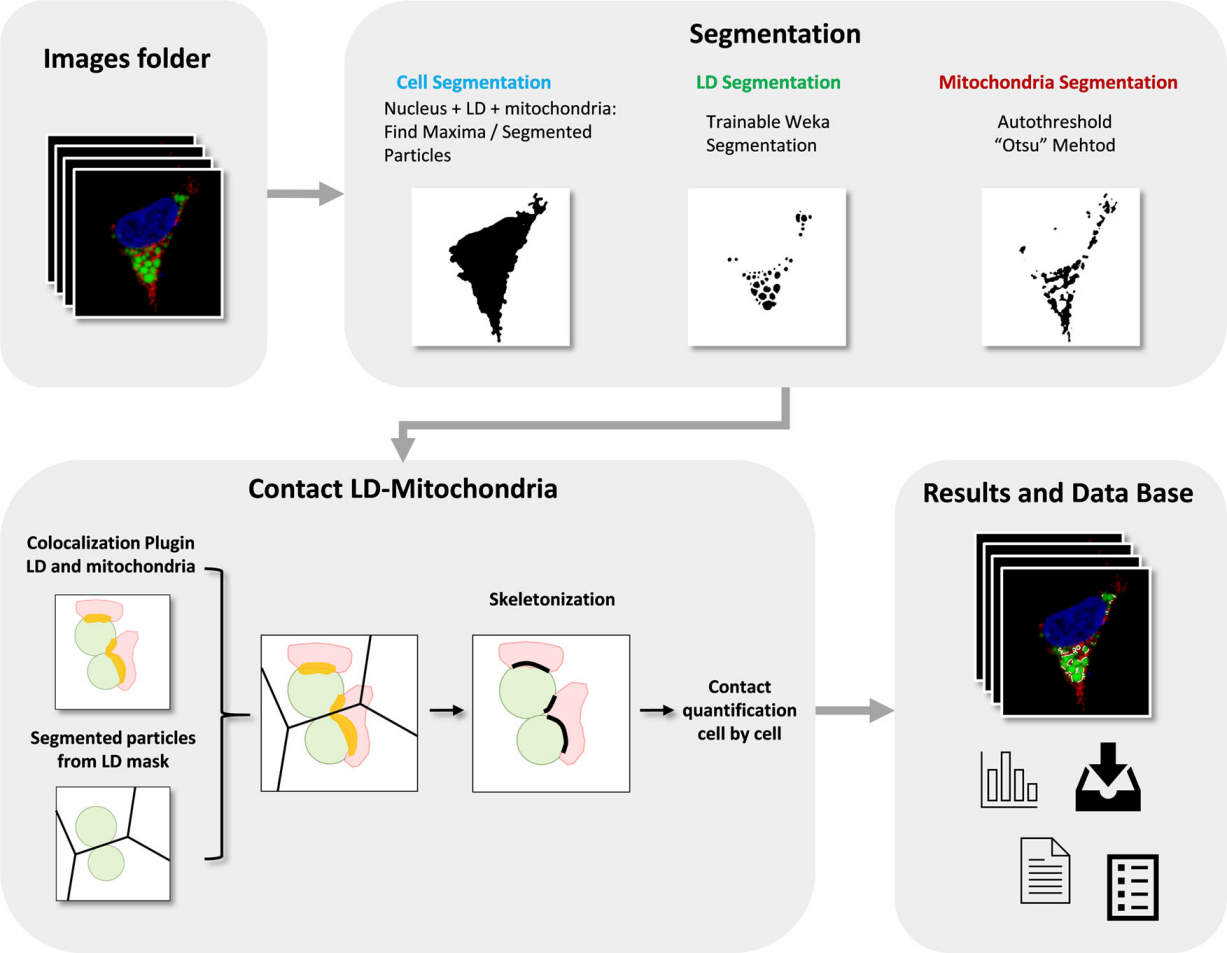
Flowchart of ContactJ Macro that automatically segments and measures the LD-mitochondria contacts cell by cell by combining standard and machine learning segmentation processes and the novel use of colocalization together with skeletonization methods from a large number of fluorescence images. LD, Mitochondria, Colocalization Area and final Contact Site are represented in green, red, yellow and black respectively.

We have developed ContactJ, a macro script for the open-source image analysis software ImageJ. ^8^^,^^9^ This macro automatically and rapidly quantifies confocal images that are saved in a folder and returns the database of the resulting measurements, images and Regions of Interests (ROIs) in a “Results” folder. Thus, inexperienced users with no prior image analysis experience will find it easy to use. As can be seen in Figure 1, the flowchart illustrates how the macro automatically detects and measures LD-mitochondria linear contacts by combining standard and machine learning segmentation processes and the novel use of colocalization together with skeletonization methods from a large number of fluorescence images.

**Figure 2.**
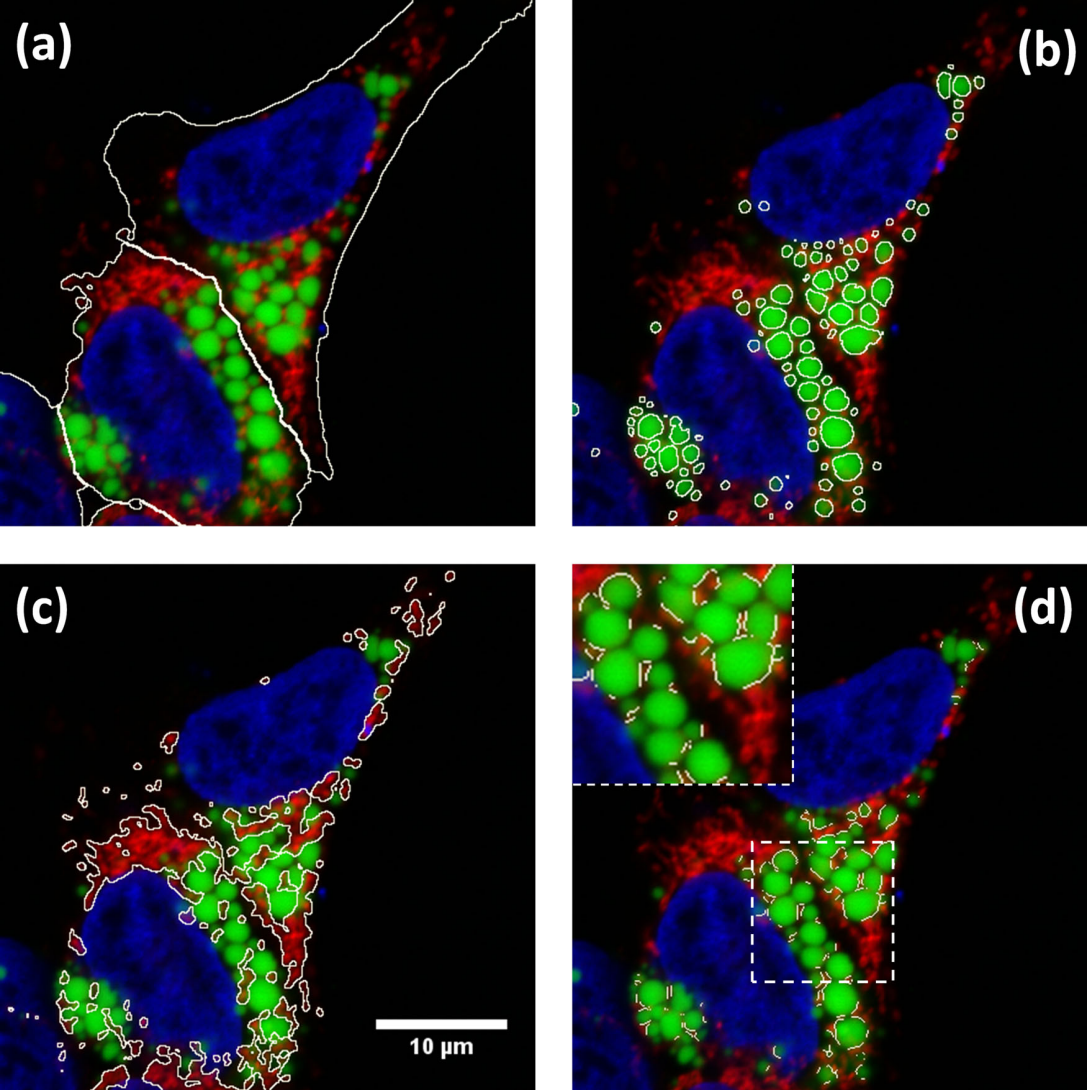
Results of ContactJ macro. Hek293 cells were labelled with anti TOM20 antibody (mitochondria) in red, Bodipy493/505 (LDs) in green and DAPI (nuclei) in blue. The different regions of interest resulting from segmentation are highlighted in white (a) Cell Segmentation, (b) LD segmentation, (c) mitochondria segmentation and (d) LD-mitochondria contacts. Insert in d) shows a detail of how contact regions found by ContactJ are accurate and individualized per LD.

First, ContactJ macro performs the segmentation of the cell, LD and mitochondria separately. For the segmentation of the cell, channels from mitochondria and LD were intensity compensated and added to a binary mask from nuclei. The resulting image was used to find local maxima (with prominence of 100) and to obtain subsequently the segmented particles binary image. Segmented particles limits were encoded as 0 value on the binarized image from the three merged channels. Limits between cells allowed to accurately segment, individualize and store cells as ROIs (see Figure 2a). LD segmentation was achieved through a Trainable Weka Segmentation classifier ^10^ on LD channel image (see Figure 2b) and mitochondria were segmented by intensity thresholding (autothreshold method “Otsu”) (see Figure 2c).

On one hand, contact regions between mitochondria and LD were first obtained using the Colocalization plugin. ^11^ This plugin highlights the colocalized “contact” points between mitochondria and LD 8-bits images (or stacks). The plugin generates an 8 bit binary image with only the colocalized points (Display value = 255). Two points are considered as colocalized if their respective intensities are strictly higher than the threshold of their channels (autothreshold methods “Yen” for LD channel and “Otsu” for mitochondria channel), and if their ratio (of intensity) is strictly higher than the ratio setting value (set to 50%: ratio (0-100%)). On the other hand, the regions of individualized LD are obtained using the Find Maxima tool with Segmented particles result from the LD mask. Finally, individualized contact regions were converted to a contour line section by performing the skeletonization of the minimum image calculation from the colocalization mask and the segmented particles result from LD. Contact perimeter and contact counts (a contact is defined as a continuous contact line) were quantified, obtaining the linear LD-mitochondria contact of each cell (see Figure 2d).

Finally, along the execution of the macro, all the data is stored in arrays (cell, LD and mitochondria areas and perimeters, contact perimeter, number of contacts, etc). Moreover, this data is stored in a.txt database file allowing the traceability of the results for each cell and each image.

### Operation

ImageJ/Fiji with the Colocalization ^11^ and WEKA ^10^ plugins should be installed and ContactJ run from ImageJ macro editor. The software can be tested with the sample data provided (in *Underlying data* ^12^). First, the user should prepare a set of images and organize them into a folder. In this images folder the user should create a subfolder named “Model” with the data and model files obtained specifically for the segmentation of LD channel using the machine learning WEKA plugin. Once ContactJ runs, macro asks to the user the folder to analyse. Automatically, ContactJ opens the images one by one analysing them, cell by cell, and saving ROIs and all the measurements data obtained (areas, intensity, contact, perimeter …) in a .txt file as a data base.

## Use cases

ContactJ has been developed and tested for the contact analysis of hundreds of HEK293 cells treated or untreated with lipopolysaccharide (LPS) and expressing or not a protein of interest PLIN5. ^4^

Taking advantage of fluorescence multilabelling, the cells have been segmented and all parameters can be expressed per cell individually. The macro segments the nucleus, LDs and mitochondria from each cell and it obtains the following parameters that are stored in a data base table: Cell Area, number of LDs and Mitochondria, LDs and Mitochondria Total Area, Mean LD Area per cell, Standard deviation of the LD mean Area, Mean LD Perimeter and Total Mitochondria and LD Perimeter

The main novelty and distinctive feature from ContactJ is that it creates a contact line corresponding to each contact site between mitochondrion and LD. In order to obtain the contact site, ContactJ first generates a colocalization region corresponding to the overlapping fluorescence from both organelles, using the Colocalization plugin. Then, this shape is skeletonized generating a line of equidistant points to its boundaries representing the contact site. The macro stores in the data base file also the total length of the contact sites, the mean length of each contact and the number of contacts detected per cell. In the mentioned work, the results were expressed as Total Contact Length/Cell and compared between cell populations and treatments. ^4^

## Discussion

One of the innovative and distinctive features of ContactJ is that it creates a linear contact region on the mid plane of the LD in close proximity to Mitochondrion, representing the most probable contact site between both organelles.

**Figure 3.**
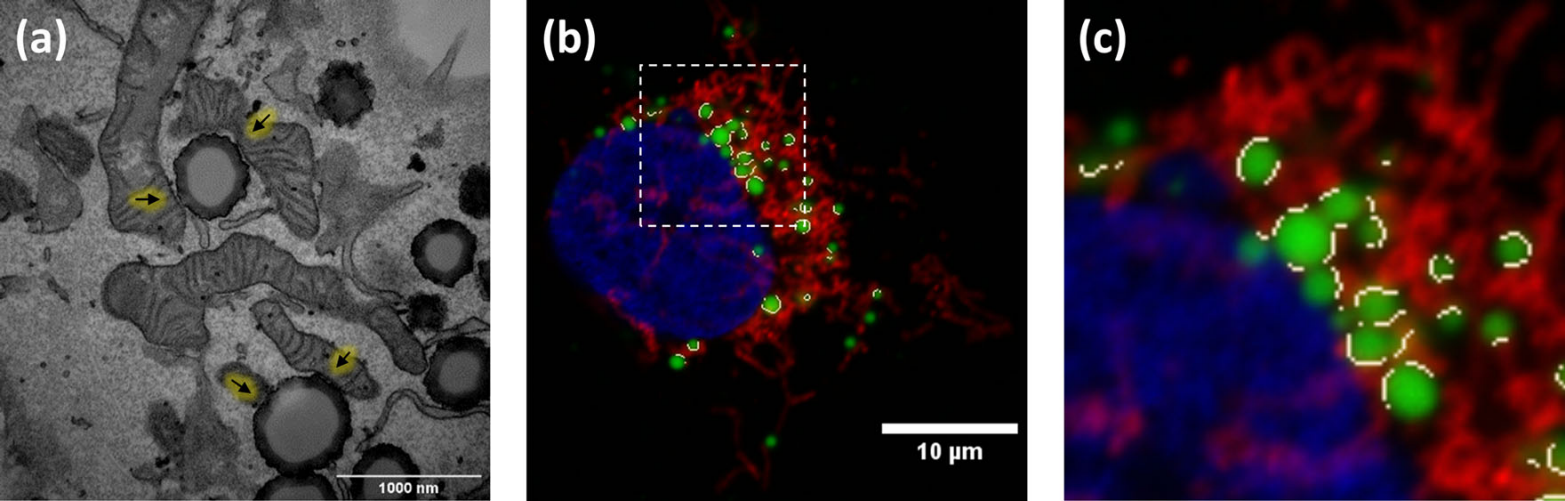
(a) TEM image showing LD-mitochondria contacts indicated by arrows, (b) result of the LD-mitochondria contacts (in white) detected by ContactJ in fluorescence microscopy image in Hek293 cells labelled with anti TOM20 antibody (mitochondria) in red, Bodipy493/505 (LDs) in green and DAPI (nuclei) in blue; and (c) magnification of the square region of (b) showing the contacts in detail.

Although light microscopy resolution limit prevents assertion of true interaction, the analysis of inter organelle contacts by fluorescence microscopy is accepted as an indicator of possible communication between these two organelles, bringing many advantages when performing contact analysis at a high scale of samples and conditions. TEM is used to measure contacts between organelles as it resolves at the membrane scale where these contacts take place, as can be seen in Figure 3(a). ContactJ measurements of contact perimeter between LD and mitochondria are 2-3 times bigger compared to TEM measurements ^4^ ( Figure 3). The main reasons for this difference are, first, that 2D confocal microscopy image represents a projection of approximately 500nm sample thickness, compared to the 70nm of the ultra-thin TEM lamella and consequently, it is collecting a higher proportion of membrane and contacts. Secondly, the intrinsic difference in resolution would affect more directly the measurements of small contacts by light microscopy overestimating them. Therefore, the contacts obtained with ContactJ can be considered reliable compared to those observed by TEM.

Obtaining contact regions, together with multiple morphological parameters of organelles, allows the calculation of descriptive statistics that would help describing cellular response. In front a metabolic or pathogenic event, cells need to regulate the transfer and communication between organelles. Among many possible cell reactions, they may change the contact surface between organelles together with the organelle size, number and distribution. For example, in this case, the quantification with Contact J of the contact length and the LD perimeter would allow the calculation of the LD-Mitochondria “transfer or communication” efficiency for each cell (Contact length/LD Perimeter Length) helping in the comparison between cells response.

In conclusion, the described image analysis workflow unveils a wide range of possibilities in the automatic quantification of LD and mitochondria contacts and it also has been tested, and it is applicable, to the study of other organelles in 2D and 3D images. Obtaining contact regions together with multiple cell and organelles parameters allow building descriptive statistics of the cells response. Moreover, its application in a large number of images enables the use of High Content Screening and Analysis, highly increasing the quality and statistical confidence of the results.

## Data availability

### Underlying data

Zenodo: UB-BioMedMicroscopy/ContactJ: ContactJ, http://doi.org/10.5281/zenodo.4569935 ^12^

This project contains the trained model and data for Weka plugin and example images.

## Software availability

Source code available from: https://github.com/UB-BioMedMicroscopy/ContactJ/tree/1.0

Archived source code as at time of publication: http://doi.org/10.5281/zenodo.4569935 ^12^

License: CC0
