# Supplementary material for: ContactJ: Characterization of lipid droplet-mitochondrial contacts using fluorescence microscopy and image analysis
Source: F1000Res. 2022 Jan 18;10:263. Originally published 2021 Apr 1. [Version 2] doi: 10.12688/f1000research.51900.2 (PMC8812330; doi:10.12688/f1000research.51900.2)
Supplement: Peer review history

# Peer review: Reviewer response for version 2

**DOI:** 10.5256/f1000research.119235.r120256

**Published:** 2022-02-02

**Authors:** Gadea Mata Martinez

**Affiliations:** 1 Department of Mathematics and Computer Science, University of La Rioja, Logroño, Spain

The authors accepted the comments made in the first revision. The new version, both of the article and the code, has improved, and I consider the article to be approved for indexing.

Are the conclusions about the tool and its performance adequately supported by the findings presented in the article?

Yes

Is the rationale for developing the new software tool clearly explained?

Yes

Is the description of the software tool technically sound?

Yes

Are sufficient details of the code, methods and analysis (if applicable) provided to allow replication of the software development and its use by others?

Partly

Is sufficient information provided to allow interpretation of the expected output datasets and any results generated using the tool?

Yes

Reviewer Expertise:

Light microscopy image analyst and computer scientist, field of artificial intelligence

I confirm that I have read this submission and believe that I have an appropriate level of expertise to confirm that it is of an acceptable scientific standard.

# Response

**Published:** 2022-02-03

**Authors:** Maria Calvo

**Affiliations:** University of Barcelona, Spain

Thanks to the reviewers Gadea Mata, Mafalda Souza and Maria Azevedo for their work reviewing the article that has helped to improve our article.

# Peer review: Reviewer response for version 2

**DOI:** 10.5256/f1000research.119235.r120255

**Published:** 2022-01-31

**Authors:** Mafalda Sousa, Maria Azevedo

**Affiliations:** 1 i3S - Instituto de Investigação e Inovação em Saúde, Universidade do Porto, Porto, Portugal; 2 3S - Instituto de Investigação e Inovação em Saúde, Universidade do Porto, Porto, Portugal

The authors accepted most of the comments made in the first revision and submitted a new version of the tool with improvements in the code and performance. At this point, we consider that the article should be approved for indexing.

Are the conclusions about the tool and its performance adequately supported by the findings presented in the article?

Partly

Is the rationale for developing the new software tool clearly explained?

Yes

Is the description of the software tool technically sound?

Yes

Are sufficient details of the code, methods and analysis (if applicable) provided to allow replication of the software development and its use by others?

No

Is sufficient information provided to allow interpretation of the expected output datasets and any results generated using the tool?

Partly

Reviewer Expertise:

Light microscopy image analysis specialist. Neuroscience.

We confirm that we have read this submission and believe that we have an appropriate level of expertise to confirm that it is of an acceptable scientific standard.

# Response

**Published:** 2022-02-03

**Authors:** Maria Calvo

**Affiliations:** University of Barcelona, Spain

Thanks to the reviewers Mafalda Souza, Maria Azevedo and Gadea Mata, for their work reviewing the article that has helped to improve our article.

# Peer review: Reviewer response for version 1

**DOI:** 10.5256/f1000research.55111.r82670

**Published:** 2021-05-24

**Authors:** Gadea Mata Martinez

**Affiliations:** 1 Department of Mathematics and Computer Science, University of La Rioja, Logroño, Spain

The authors present ContactJ, an ImageJ macro, to identify and quantify contacts between lipid droplets (LD) and mitochondria.

 This type of analysis is usually performed using electron microscopy. The authors have found a way to carry out a similar study (taking into account the limitations of resolution) using confocal microscopy images.

 The use of this type of image along with the combination of different analytical techniques and machine learning make this macro an interesting and novel work.

 The authors clearly describe the workflow of the macro. I would like to highlight the effort that has been put into establishing a clear distinction between each part of it, which helps to monitor and check the results at all times. The figures shown are very clear and leave no room for doubt. Another strength of this work is that this macro has already been used to analyse the work done in a publication.

 However, I want to comment on some minor aspects that could be subject to improvement:

 Note that the link in reference 11 is broken. Please replace with:

https://imagej.nih.gov/ij/plugins/colocalization.html

 In the Implementation section, the macro configuration is clearly defined and the parameters that have been used in each step are well described. However, the type of machine learning algorithm used for the LD segmentation is missing.

 In the Discussion section, it is mentioned that the study can be carried out for 3D images. Adding an example of this type in the test images file is recommended.

 In this same section, the authors mention that this macro can be used to analyse a large number of images (High Content Screening and Analysis). For this type of analysis, it would be helpful that the option to check if the cells are well-segmented could be activated or deactivated. This way, it could be used to check some images and then run the macro over the entire experiment unsupervised.

 The macro is fast, and its code is well commented, which makes it easy to read and understand. Considering the different domains that a single type of image may have (such as changes in the intensity of the sample or noise), I suggest that the configuration parameters of the different methods become variables that are described at the beginning of the macro. Therefore, the macro gives the user the possibility, if needed, to quickly and easily change the parameters, avoiding changes within the code.

 Similarly, in addition to converting the channels used each time into a variable, it is recommended to comment on the code and refer to the channels not only by their colour but also by what they contain, as sometimes the experts use different criteria for the colour that each channel represents.

 As the macro progresses, the user can watch how several images are opened, which represent the intermediate steps. This intermediate image display slows down the macro, and it is suggested to use the “setBatchMode (true)” option whenever possible to avoid this effect.

 In addition, it is observed that the intermediate images that have been generated in the different steps remain open until the final result is obtained. To improve the performance of the macro, it is recommended that they are automatically closed when they are no longer needed.

 Regarding the use of GitHub to upload the macro, I recommend that the authors complete the README section, providing information on the installation of the macro, the requirements that are needed for the correct functioning of the macro, and a brief description of its use.

 The macro gives a robust set of results, which offer the possibility of obtaining interesting relationships, as the authors have commented. However, I recommend adding the calculation of the standard deviation for both the mean of the perimeter of the LD and the mean of the length of the Contact. Since both means are already calculated, it is convenient to calculate the standard deviations for each one.

 All this would improve and facilitate the use of this macro by others in a general, simple and clear way.

Are the conclusions about the tool and its performance adequately supported by the findings presented in the article?

Yes

Is the rationale for developing the new software tool clearly explained?

Yes

Is the description of the software tool technically sound?

Yes

Are sufficient details of the code, methods and analysis (if applicable) provided to allow replication of the software development and its use by others?

Partly

Is sufficient information provided to allow interpretation of the expected output datasets and any results generated using the tool?

Yes

Reviewer Expertise:

Light microscopy image analyst and computer scientist, field of artificial intelligence

I confirm that I have read this submission and believe that I have an appropriate level of expertise to confirm that it is of an acceptable scientific standard, however I have significant reservations, as outlined above.

# Response

**Published:** 2021-12-31

**Authors:** Maria Calvo

**Affiliations:** University of Barcelona, Spain

**The authors present ContactJ, an ImageJ macro, to identify and quantify contacts between lipid droplets (LD) and mitochondria.**

**This type of analysis is usually performed using electron microscopy. The authors have found a way to carry out a similar study (taking into account the limitations of resolution) using confocal microscopy images.**

**The use of this type of image along with the combination of different analytical techniques and machine learning make this macro an interesting and novel work.**

**The authors clearly describe the workflow of the macro. I would like to highlight the effort that has been put into establishing a clear distinction between each part of it, which helps to monitor and check the results at all times. The figures shown are very clear and leave no room for doubt. Another strength of this work is that this macro has already been used to analyse the work done in a publication.**

**However, I want to comment on some minor aspects that could be subject to improvement:**

*Thank you for reviewing the article and pointing out the issues that would help to improve the presented work. We have tried to address all the suggestions and criticisms.*

**1) Note that the link in reference 11 is broken. Please replace with:**

**https://imagej.nih.gov/ij/plugins/colocalization.html**

*Thank you for the clarification, we have corrected it. After the review we have noticed that this plugin is not stable* *in new FIJI versions. In order to avoid it we have added the code that allows to obtain the colocalization mask (based from the reference Bourdoncle https://imagej.nih.gov/ij/plugins/colocalization.html). This specific code was previously adapted to analyse colocalization with no restrictions of bit depth and for stacks (Vidal-Quadras et al. Traffic. 2011 Dec;12(12):1879-96. doi: 10.1111/j.1600-0854.2011.01274.x. Epub 2011 Sep 21.)*

**2) In the Implementation section, the macro configuration is clearly defined and the parameters that have been used in each step are well described. However, the type of machine learning algorithm used for the LD segmentation is missing.**

*In agreement with the referee, the information about the Machine Learning algorithms used for LD segmentation is provided and included in the manuscript (methods section).*

*Changes in the manuscript:*

*“LD segmentation was achieved through a Trainable Weka Segmentation classifier on LD channel image (see Figure 2b)) using the classification algorithm Fast Random Forest and the following Training features: Gaussian Blur, Hessian, Membrane projections, Sobel filtre, Difference of Gaussians. Mitochondria were segmented by intensity thresholding (autothreshold method “Otsu”)*

**3) In the Discussion section, it is mentioned that the study can be carried out for 3D images. Adding an example of this type in the test images file is recommended.**

*As mentioned throughout the work, contact analysis with ContactJ can be adapted to any image type i.e. 3D images (xyz) or through time (xyt). However, the direct purpose of this macro is the analysis of* *hundreds of cells and multiple conditions from 2D images and therefore it has been optimized for this type of images.*

*As suggested by the referees, in order to adapt contact analysis to any 3D objects analysis or objects tracking methods, we have developed a new multi image ContactJ code that generates a* *contacts mask from two channels* *from images either 2D or from stacks (3D or time series). This code is available at github, open to all kind of projects and ready to be included in any code workflow. Also an example dataset from xyz and xyt images is included (* *https://zenodo.org/badge/latestdoi/442839907* *).*

**4) In this same section, the authors mention that this macro can be used to analyse a large number of images (High Content Screening and Analysis). For this type of analysis, it would be helpful that the option to check if the cells are well-segmented could be activated or deactivated. This way, it could be used to check some images and then run the macro over the entire experiment unsupervised.**

*We agree that this option will be helpful for checking performance of the macro and it has been included at the GUI at the beginning of the macro.*

**5) The macro is fast, and its code is well commented, which makes it easy to read and understand. Considering the different domains that a single type of image may have (such as changes in the intensity of the sample or noise), I suggest that the configuration parameters of the different methods become variables that are described at the beginning of the macro. Therefore, the macro gives the user the possibility, if needed, to quickly and easily change the parameters, avoiding changes within the code.**

**This option can be selected at GUI at the beginning of the macro.**

*As suggested by the referees, an initial GUI has been implemented to define the image dependent variables (thresholds, ratio…).*

**6) Similarly, in addition to converting the channels used each time into a variable, it is recommended to comment on the code and refer to the channels not only by their colour but also by what they contain, as sometimes the experts use different criteria for the colour that each channel represents.**

*We agree that referring to the channels by what they represent will be more self-explanatory.*

**7) As the macro progresses, the user can watch how several images are opened, which represent the intermediate steps. This intermediate image display slows down the macro, and it is suggested to use the “setBatchMode (true)” option whenever possible to avoid this effect.**

*We totally agree that setting the macro to Batch Mode will fasten its performance, therefore we have set it.*

**8) In addition, it is observed that the intermediate images that have been generated in the different steps remain open until the final result is obtained. To improve the performance of the macro, it is recommended that they are automatically closed when they are no longer needed.**

*To improve the code’s performance, now images are being closed when not necessary.*

**9) Regarding the use of GitHub to upload the macro, I recommend that the authors complete the README section, providing information on the installation of the macro, the requirements that are needed for the correct functioning of the macro, and a brief description of its use.**

*We have provided the requested information in the Readme section of GitHub.*

**10) The macro gives a robust set of results, which offer the possibility of obtaining interesting relationships, as the authors have commented. However, I recommend adding the calculation of the standard deviation for both the mean of the perimeter of the LD and the mean of the length of the Contact. Since both means are already calculated, it is convenient to calculate the standard deviations for each one. All this would improve and facilitate the use of this macro by others in a general, simple and clear way.**

*We totally agree that this data was missing and its inclusion improves the obtained information.*

# Peer review: Reviewer response for version 1

**DOI:** 10.5256/f1000research.55111.r82672

**Published:** 2021-04-22

**Authors:** Mafalda Sousa, Maria Azevedo

**Affiliations:** 1 i3S - Instituto de Investigação e Inovação em Saúde, Universidade do Porto, Porto, Portugal; 2 3S - Instituto de Investigação e Inovação em Saúde, Universidade do Porto, Porto, Portugal

A novel ImageJ macro script called ContactJ that allows for the automatic determination of contacts formed between lipid droplets (LD) and mitochondria in cells is presented. An interaction that is usually assessed by electron microscopy, involving a reduced number of biological events analyzed, is assessed in this work by the combination of fluorescence microscopy with automatic image analysis. This approach boosts the number of biological events analyzed. It turns the analysis more time-efficient by using colocalization methods combined with the segmentation of the LD, which extracts the contact region on the midplane of the LD that is in close proximity of mitochondria.

 The limitation of using light microscopy to look at very small contacts is clearly stated. Nevertheless, the use of this imaging technique as an indicator of possible contact between different organelles is well accepted in the community. Light microscopy was already shown to provide more information about the contacts between LD and mitochondria due to the sample thickness that is bigger than the sample prepared for transmission electron microscopy.

 ContactJ is presented as a well-established workflow. The authors combine tools previously developed in an exquisite way, such as the trainable Weka segmentation classifier and the colocalization plugin, with the conventional object segmentation. Figure 1 is very self-explanatory of the workflow followed by the macro script. Another positive aspect is the detailed output of the macro script that allows for a complex and quantitative analysis of the contacts established between the LD and mitochondria. The *Operation *section description is well written, and no problems were found while running the macro script; it is a very simple and straightforward process.

 Despite the positive aspects mentioned above, there are fewer positive points that need to be checked:

 Regarding the strategy applied for the segmentation of the cells (page 3), most probably extra cell labeling could be used to facilitate this step. Usually, the staining of a cytoskeleton protein or the cell membrane staining is used to define the cells' boundary facilitating the cell segmentation step. The authors may consider such an approach in the sample preparation and imaging.

 Not so thorough is the description of the code behind the workflow. The authors are sparse in describing the different steps that make up the workflow. Some of those steps are not even mentioned in the *Implementation *section of the manuscript, being only perceptible by reading or running the code line by line, which jeopardizes reproducibility. Likewise, the comments along the macro script are also sparse. A more detailed and extensive workflow description is needed, both in the manuscript and in the macro script. ImageJ good code practices suggest that you mention the ImageJ version and cite it in the associated publication.

 The citation " *channels from mitochondria and LD were intensity compensated*" should be explained to be more explicit. The authors commented in the script (line 143) "calculate addition 0.2LD+0.2mitochondria", meaning that the intensity compensation was 20% of the original values. Is there a reason for assigning this value? Should it be considered as a variable dependent on the dataset?

 In the *Implementation *section (page 4), it is stated that “ *the colocalized "contact" points between the mitochondria and LD 8-bit images (or stacks)*". It is not clear if an 8-bit image is mandatory for determining the contact points or the number of channels that may compose the image. These details should be clearly stated. Additionally, as the reference to use a stack of images is made, the dataset available to test the macro script should contain this type of image.

 The available dataset for testing the code is unfortunately diminished. Only two very similar images are available for testing the script. The dataset for testing this tool should be bigger and the images must represent a real, heterogeneous population of cells to test the proposed script’s execution and performance.

 Following the same lines, the script should be tested for quality and performance and presented in the manuscript. A previous publication shows the analysis of hundreds of HEK293 cells by the application of ContactJ. However, a comparison between the ContactJ results and another analysis method (such as manual quantification or using proprietary software) is not presented. Moreover, the usage of ContactJ to look at LD-mitochondria contacts in different cells (primary cells such as neurons, or other cell lines such as PC12 or CAD cells) could be included. Such validation is of extreme importance to show robustness and accuracy on the data obtained by the application of ContactJ.

 Throughout the manuscript, the term *database* refers to the script output .txt file created upon the analysis (page 5). In our understanding, this terminology is incorrectly used since a database is a set of related tables. The authors can use the term *table *since the output is indeed a table.

 Looking closer to the presented macro in ImageJ, some adjustments should be made. Along with the code, several hard-coded values are used, such as thresholding values and filter parameters. It is unclear how these values may be applied in different images or conditions. Maybe the authors should consider building a graphical user interface for prompting these parameters. Plus, there is no code error handling, not testing, for example, if the input is an 8-bit image with 3 channels. The algorithm premises that images are 8-bit depth, although this is not mentioned nor guaranteed along with the code.

 There is likely an error in the option of removing cell ROIs (lines 180-190). When deleting the cells, the results table with cell measurements is not updated and the deleted ROIs are the last ones on the table and not the ones corresponding to the correct position in the image. This error affects the results obtained, so it should be corrected. Furthermore, the *Resulting txt file *needs to be truncated or deleted from the results folder. Otherwise, it will append the results of further analysis.

 Running the ContactJ macro script takes some time (this should also be measured). The authors could improve the code performance by reducing the number of *for*-cycles and by closing the images while the code is running (not only at the end). Also, the analysis could be set in batch mode (true), showing only the images for quality control.

 While ContactJ is being presented as a tool to look for contacts between LD and mitochondria, the translational application of this script is not obvious. It might strengthen the work if more broad applicability of the ContactJ is mentioned or, at least, speculated. We see the potential for the application of ContactJ on image analysis where interaction between different cell types (oligodendrocytes-neuron interaction, microglia-neuron interaction, yeast-macrophage interaction.), or between different cellular organelles is done. May the authors comment on these possible applications and add them to the Discussion section?

Are the conclusions about the tool and its performance adequately supported by the findings presented in the article?

Partly

Is the rationale for developing the new software tool clearly explained?

Yes

Is the description of the software tool technically sound?

Yes

Are sufficient details of the code, methods and analysis (if applicable) provided to allow replication of the software development and its use by others?

No

Is sufficient information provided to allow interpretation of the expected output datasets and any results generated using the tool?

Partly

Reviewer Expertise:

Light microscopy image analysis specialist. Neuroscience.

We confirm that we have read this submission and believe that we have an appropriate level of expertise to confirm that it is of an acceptable scientific standard, however we have significant reservations, as outlined above.

# Response

**Published:** 2021-12-31

**Authors:** Maria Calvo

**Affiliations:** University of Barcelona, Spain

**A novel ImageJ macro script called ContactJ that allows for the automatic determination of contacts formed between lipid droplets (LD) and mitochondria in cells is presented. An interaction that is usually assessed by electron microscopy, involving a reduced number of biological events analyzed, is assessed in this work by the combination of fluorescence microscopy with automatic image analysis. This approach boosts the number of biological events analyzed. It turns the analysis more time-efficient by using colocalization methods combined with the segmentation of the LD, which extracts the contact region on the midplane of the LD that is in close proximity of mitochondria.**

**The limitation of using light microscopy to look at very small contacts is clearly stated. Nevertheless, the use of this imaging technique as an indicator of possible contact between different organelles is well accepted in the community. Light microscopy was already shown to provide more information about the contacts between LD and mitochondria due to the sample thickness that is bigger than the sample prepared for transmission electron microscopy.**

**ContactJ is presented as a well-established workflow. The authors combine tools previously developed in an exquisite way, such as the trainable Weka segmentation classifier and the colocalization plugin, with the conventional object segmentation. Figure 1 is very self-explanatory of the workflow followed by the macro script. Another positive aspect is the detailed output of the macro script that allows for a complex and quantitative analysis of the contacts established between the LD and mitochondria. The *Operation *section description is well written, and no problems were found while running the macro script; it is a very simple and straightforward process.**

**Despite the positive aspects mentioned above, there are fewer positive points that need to be checked:**

*Thank you for reviewing the article and pointing out the issues that would help to improve the presented work. We have tried to address all the suggestions and criticisms.*

**1) Regarding the strategy applied for the segmentation of the cells (page 3), most probably extra cell labelling could be used to facilitate this step. Usually, the staining of a cytoskeleton protein or the cell membrane staining is used to define the cells' boundary facilitating the cell segmentation step. The authors may consider such an approach in the sample preparation and imaging.**

*The cell segmentation has been adapted to a specific sample and experimental conditions that didn’t support any cell mask or cytoskeleton labelling. To clarify this point, we have added a comment in the methods section that whenever is possible it is recommended to add a fourth channel of cytoskeleton, membrane or cell labelling to facilitate the cell segmentation step.*

*Changes in the manuscript:*

*“The present work has been adapted to the particular conditions of this experiment dataset where membrane or cell mask labelling couldn’t be implemented. For this reason, a smart strategy for the cell segmentation has been used: the addition of the available cytoplasmic labellings (LD plus Mitochondria) and the Nucleus staining to define the cell region. Whenever it is possible, it is recommended to use a cell mask labelling to facilitate cell segmentation and individualization. In such case, the workflow for cell segmentation should be modified.”*

**2) Not so thorough is the description of the code behind the workflow. The authors are sparse in describing the different steps that make up the workflow. Some of those steps are not even mentioned in the *Implementation *section of the manuscript, being only perceptible by reading or running the code line by line, which jeopardizes reproducibility. Likewise, the comments along the macro script are also sparse. A more detailed and extensive workflow description is needed, both in the manuscript and in the macro script. ImageJ good code practices suggest that you mention the ImageJ version and cite it in the associated publication.**

*We have added a more detailed and extensive workflow description and the ImageJ version both in the manuscript and in the macro script.*

**3) The citation " *channels from mitochondria and LD were intensity compensated*" should be explained to be more explicit. The authors commented in the script (line 143) "calculate addition 0.2LD+0.2mitochondria", meaning that the intensity compensation was 20% of the original values. Is there a reason for assigning this value? Should it be considered as a variable dependent on the dataset?**

*As previously explained, the cell segmentation was adapted to the particular conditions of this experiment dataset where membrane or cell mask labelling couldn’t be implemented. In this case, a practical strategy was to use the addition of the available cytoplasmic labelling (LD plus Mitochondria) and the Nucleus staining to define the cell region. In such particular case, in order to have similar intensity contributions from cytoplasmic labelling and nucleus, LD and mitochondria channels were multiplied by 0.2 to reduce its intensity contribution and make it more similar to the nucleus intensity.*

*As pointed by the reviewers this value is dependent on the dataset and we have included a GUI to change this and other dataset dependent parameters*.

*Changes in the manuscript:*

*“ First, ContactJ macro performs the segmentation of the cells, LD and mitochondria separately. For the segmentation of the cell, channels from mitochondria and LD were intensity compensated and added to a binary mask from nuclei. Cell segmentation was adapted to the particular conditions of this experiment dataset where a membrane or a cell mask labelling couldn’t be implemented. Briefly, cell segmentation was achieved by adding the available cytoplasmic labellings (LD plus Mitochondria) and the Nucleus staining. To obtain similar intensity contributions from cytoplasmic labelling and nucleus, LD and mitochondria channels were multiplied each by 0.2 to reduce its intensity contribution and make it more similar to the nucleus intensity. As these factors are dependent on the different experimental conditions, they can be modified at the graphical user interface (GUI) at the beginning of the macro (referred at the GUI as Compensation). Intensity compensated channels from mitochondria and LD were added to a binary mask from nuclei.”*

**4) In the *Implementation *section (page 4), it is stated that “ *the colocalized "contact" points between the mitochondria and LD 8-bit images (or stacks)*". It is not clear if an 8-bit image is mandatory for determining the contact points or the number of channels that may compose the image. These details should be clearly stated. Additionally, as the reference to use a stack of images is made, the dataset available to test the macro script should contain this type of image.**

*As suggested by the referees, a more detailed description of the input image requirements has been included in the manuscript and the macro script.*

*Changes in the manuscript:*

*“This macro automatically and rapidly quantifies confocal images (1 section, 8-12-16 bit depth)”*

*In reference to the stack images option, there is no limitation to implement the analysis through 3D stacks or time series, however the direct purpose of this macro was the analysis of 2D images* *to extend contact analysis to hundreds of cells and multiple conditions* *.*

*As mentioned along the work, contact analysis can be extended in any image type i.e. 3D images or through time. To facilitate this, we provide, at github, the core code of ContactJ (ContactJ4All) that generates a contacts mask from two channels from 2D images or from stacks (3D or time series). By combining the core ContactJ code with 3D objects analysis or objects tracking, contact analysis can be extended in 3D or time series.*

*Changes in the manuscript:*

*“ ContactJ application can be extended to the contact analysis between other organelles, in 2D, 3D or time series. In fact, this code has been already adapted for the contact tracking analysis between LD and Endoplasmic Reticulum in time lapse experiments (13). To facilitate the applicability of ContactJ in 3D or time series images, and its combination with any available 3D objects analysis or objects tracking methods, respectively, a straightforward core code of ContactJ (ContactJ4All) has been developed. This code is ready to be included in any workflow and available on github https://zenodo.org/badge/latestdoi/442839907* *. Also, an example dataset from xyz and xyt images is included to test its performance.”*

***5) *The available dataset for testing the code is unfortunately diminished. Only two very similar images are available for testing the script. The dataset for testing this tool should be bigger and the images must represent a real, heterogeneous population of cells to test the proposed script’s execution and performance.**

*To test the script performance and reproducibility, the dataset has been increased with more images representing a heterogeneous population of cells.*

**6) Following the same lines, the script should be tested for quality and performance and presented in the manuscript. A previous publication shows the analysis of hundreds of HEK293 cells by the application of ContactJ. However, a comparison between the ContactJ results and another analysis method (such as manual quantification or using proprietary software) is not presented. Moreover, the usage of ContactJ to look at LD-mitochondria contacts in different cells (primary cells such as neurons, or other cell lines such as PC12 or CAD cells) could be included. Such validation is of extreme importance to show robustness and accuracy on the data obtained by the application of ContactJ.**

*As suggested by the referee, a comparison between the ContactJ results and manual quantification has been performed. The comparison between ContactJ analysis vs Manual analysis shows that although both methods correlate, manual analysis-increases significantly standard deviation reducing reproducibility of the method and overestimating contact length.*

*Additionally, while comparing both methods we have experienced that manual quantification doesn’t allow precise contact tracing around organelles, secondly, that it’s difficult to stablish visual criteria of proximity and finally, that manual tracing is not only time consuming but, after defining tens of contacts per cell, it results in quick user fatigue and loss of detection and precision. All these results reinforce the idea that ContactJ is an objective and reproducible method valid to define and quantify contacts between organelles automatically.”*

*Furthermore, robustness of ContactJ has been tested and validated also in contact analysis between Endoplasmic Reticulum and Lipid Droplets in U2OS cells in live cell imaging experiments* https://doi.org/10.1083/jcb.202105060).

*Changes in the manuscript:*

*“In order to test quality and performance of ContactJ, a comparison between contact manual tracing analysis compared to ContactJ analysis has been performed on 20 cells. Mean and Standard Deviation (microns) from total contact length from Manual quantification were: 44.9220 and 30.1140, respectively, and from ContactJ analysis were: 30.8055 and 18.30792, respectively. ANOVA test was performed and showed no significant difference between both groups means (F=3.209 and p= 0.08) B). Correlation Analysis of the values obtained with both methods showed a correlation index of 0.899 significant at the 0.01 level (see extended data* *Manual_vs_ContactJ_methods.pdf* *).*

*The comparison between ContactJ analysis vs Manual analysis shows that although both methods correlate, manual analysis-increases significantly standard deviation reducing reproducibility of the method and overestimating contact length.*

*Additionally, while comparing both methods we have experienced that manual quantification doesn’t allow precise contact tracing around organelles, secondly, that it’s difficult to stablish visual criteria of proximity and finally, that manual tracing is not only time consuming but, after defining tens of contacts per cell, it results in quick user fatigue and loss of detection and precision. All these results reinforce the idea that ContactJ is an objective and reproducible method valid to define and quantify contacts between organelles automatically.”*

**7) Throughout the manuscript, the term *database* refers to the script output .txt file created upon the analysis (page 5). In our understanding, this terminology is incorrectly used since a database is a set of related tables. The authors can use the term *table *since the output is indeed a table.**

*We totally agree that the term database should be substituted by the term table. It has been corrected in the manuscript.*

*Changes in the manuscript:*

*“...that are saved in a folder and returns the table of the resulting measurements, images and Regions of Interests (ROIs) in a “Results” folder.”*

**8) Looking closer to the presented macro in ImageJ, some adjustments should be made. Along with the code, several hard-coded values are used, such as thresholding values and filter parameters. It is unclear how these values may be applied in different images or conditions. Maybe the authors should consider building a graphical user interface for prompting these parameters. Plus, there is no code error handling, not testing, for example, if the input is an 8-bit image with 3 channels. The algorithm premises that images are 8-bit depth, although this is not mentioned nor guaranteed along with the code.**

*An initial GUI has been implemented to define the dataset dependent parameters (thresholds, ratio…).*

*A more accurate description of the input image requirements has been included (bit depth, channels…) in the manuscript and the macro script.*

**9) There is likely an error in the option of removing cell ROIs (lines 180-190). When deleting the cells, the results table with cell measurements is not updated and the deleted ROIs are the last ones on the table and not the ones corresponding to the correct position in the image. This error affects the results obtained, so it should be corrected. Furthermore, the *Resulting txt file *needs to be truncated or deleted from the results folder. Otherwise, it will append the results of further analysis.**

*The reported error has been fixed and now the deleted ROIs results are properly removed from the generated results table.*

**10) Running the ContactJ macro script takes some time (this should also be measured). The authors could improve the code performance by reducing the number of *for*-cycles and by closing the images while the code is running (not only at the end). Also, the analysis could be set in batch mode (true), showing only the images for quality control.**

*The code has been reviewed to avoid redundant operations. Images now, are being closed when not necessary to improve the code’s performance. In addition, the analysis has been set in batch mode.*

**11) While ContactJ is being presented as a tool to look for contacts between LD and mitochondria, the translational application of this script is not obvious. It might strengthen the work if more broad applicability of the ContactJ is mentioned or, at least, speculated. We see the potential for the application of ContactJ on image analysis where interaction between different cell types (oligodendrocytes-neuron interaction, microglia-neuron interaction, yeast-macrophage interaction.), or between different cellular organelles is done. May the authors comment on these possible applications and add them to the Discussion section?**

*The translational application of ContactJ is clear in the context of the lipid droplets role because their interaction with other organelles are now recognized as key factors in physiological and disease processes. As suggested by the referees we have commented the translational aspect of the macro and its possible applications in the discussion.*

*We appreciate the referees view of ContactJ ‘s potential. In fact, this code has already been extended to the contact analysis between LD and Endoplasmic Reticulum in time lapse experiments (A. Lu et al.* *J. Cell Biol. 2021 Vol. 221 No. 2 e202105060* *https://doi.org/10.1083/jcb.202105060* *).*

*To facilitate the applicability of ContactJ in 3D or time series images, and its combination with any available 3D objects analysis or objects tracking methods, respectively, we have developed a straightforward core code of ContactJ (ContactJ4all). This code is ready to be included in any workflow and available on github. Also, an example dataset from xyz and xyt images is included to test its performance* *https://zenodo.org/badge/latestdoi/442839907*
